# Variable selection for inferential models with relatively high-dimensional data: Between method heterogeneity and covariate stability as adjuncts to robust selection

**DOI:** 10.1038/s41598-020-64829-0

**Published:** 2020-05-14

**Authors:** Eliana Lima, Peers Davies, Jasmeet Kaler, Fiona Lovatt, Martin Green

**Affiliations:** 1 School of Veterinary Medicine and Science, University of Nottingham, Sutton Bonington Campus, Leicestershire, LE12 5RD United Kingdom; 2 Department of Epidemiology and Population Health, Institute of Infection and Global Health, University of Liverpool, Liverpool, L69 7BE United Kingdom; 3 Present Address: OIE, World Organisation for Animal Health 12, rue de Prony, 75017 Paris, France

**Keywords:** Computational biology and bioinformatics, Risk factors, Mathematics and computing

## Abstract

Variable selection in inferential modelling is problematic when the number of variables is large relative to the number of data points, especially when multicollinearity is present. A variety of techniques have been described to identify ‘important’ subsets of variables from within a large parameter space but these may produce different results which creates difficulties with inference and reproducibility. Our aim was evaluate the extent to which variable selection would change depending on statistical approach and whether triangulation across methods could enhance data interpretation. A real dataset containing 408 subjects, 337 explanatory variables and a normally distributed outcome was used. We show that with model hyperparameters optimised to minimise cross validation error, ten methods of automated variable selection produced markedly different results; different variables were selected and model sparsity varied greatly. Comparison between multiple methods provided valuable additional insights. Two variables that were consistently selected and stable across all methods accounted for the majority of the explainable variability; these were the most plausible important candidate variables. Further variables of importance were identified from evaluating selection stability across all methods. In conclusion, triangulation of results across methods, including use of covariate stability, can greatly enhance data interpretation and confidence in variable selection.

## Introduction

Variable selection is an integral and critical component of inferential modelling and methods to accurately detect the subset of variables most likely to have true associations with an outcome of interest are essential. In observational or experimental research, when potential causal covariates need to be identified to be carried forward for future study, a very large set of variables may need to be explored and a robust method is required to identify the most likely candidate covariates from within a large parameter data space.

Identification of a best subset of variables is known to be problematic when the number of explanatory variables is large with respect to the number of subjects and when multicollinearity is present within the data^1^. In this situation, despite their widespread use, it is recognised that selection methods based on exploratory or stepwise procedures using P-values or likelihood-based methods have notable deficiencies including producing inflated coefficient estimates and downward biased errors^1–4^. This generally results in models that are over fit and with a relatively high number of variables remaining in a ‘final’ model rather than a sparse model that contains only variables with the greatest association with the outcome^1^. The sparsity principle (that a relatively small number of predictors contribute meaningfully to the response), is commonly adopted for variable selection of high dimensional data and substantial research has been conducted into selection of sparse models from high dimensional data^3^. General approaches to variable selection have been reviewed and available methods described under three main categories; test-based, penalty-based and screening-based^5^. It has also been noted that improvements to robust variable selection can be made through the use of selection stability^6,7^. The rationale for this is that, since model selection procedures generally suffer from inconsistency, resampling is used to evaluate the extent to which selected covariates change when the data are randomly divided or perturbed^8–10^. Stable variables (i.e. those selected most consistently under subsampling) are most likely to have a true association with the outcome of interest in a target population and are therefore most likely to be good candidates to evaluate further.

Although a variety of techniques for covariate selection have been advocated^11–14^, since different techniques have different mathematical properties, it is possible they will lead to different solutions; that is different variables may be selected. A current problem for the researcher is to know the extent to which the choice of method of selection will impact upon study results and therefore, the extent to which results will be reproducible with different methods. Therefore, another area in which variability can be explored is ‘between-method’ variability in covariate selection. Indeed it has been suggested that good practice is to employ a variety of methods “and assign the degree of confidence to variables depending on how many methods selected a particular variable in the final subset”^15^. Results that are triangulated using different methods are considered less likely to be artefacts^16^.This sentiment is also expressed in the recent concerns related to use of P values to identify important variables in which it has been suggested that researchers have ‘the courage to consider uncertainty from multiple angles in every study’ and ‘analyse data in multiple ways to see whether different analyses converge on the same answer’^17^. Such triangulation, however, is rarely conducted.

The aim of this research was to evaluate a relatively high dimensional observational animal production-based dataset to compare results obtained from ten well-documented, automated methods of variable selection. The purpose was to identify the extent to which variable selection would change depending on method used and whether combining results across methods could provide additional insights into the selection process.

## Results

### Covariates selected in the final models

The outcome of interest was the financial income derived from lamb sales on 408 UK sheep farms in 2017 (£/acre) and the comparison between ten automated methods of variable selection revealed substantial differences between covariates selected in final models. The methods used were backward stepwise linear regression (BSLM), multivariate adaptive regression splines (MARS), least absolute shrinkage and selection operator regression (lasso), ridge regression (ridge), elastic net regression (enet), adaptive elastic net regression (Aenet), smoothly clipped absolute deviation (SCAD), minimax convex penalty (MCP), Sparsestep, and ranking-based variable selection (RBVS). The numbers of variables selected using each method, are summarised in Table 1. Between 335 (ridge) and 2 (MARS) covariates were selected in the final models and five methods produced relatively sparse models with ≤5 variables being selected. In terms of model fit, the internal and cross validation mean absolute error (MAE) and R^2^ for all final models are displayed in Table 1. The best cross validation MAE was achieved using RBVS (64.1), closely followed by lasso and elastic net (64.6). All other models had a cross validation MAE ≤ 80.6 with the exception of BLSM, which performed poorly (MAE = 136.0). Overfitting, demonstrated by a large difference between internal and cross validation R^2^ values was most apparent in the BSLR (0.95 vs 0.28) and ridge (0.78 vs 0.57) models. Under fitting, shown by relatively low values for both internal and cross validation R^2^ values compared to the best models, was most evident in the MARS (0.67 and 0.56) and Aenet (0.68 and 0.58) models. The three sparsest models (with ≤ 5 covariates) that maintained a reasonable performance (cross validation MAE < 73.0) were MCP, SparseStep and RBVS. It was notable that models both with very few covariates (e.g. RBVS; 5 variables MAE = 64.1, MCP; 3 variables MAE = 67.4) or a larger number (e.g. SCAD; 19 variables MAE = 65.4, elastic net; 42 variables MAE = 64.6) could result in a similarly low cross validation error.

**Table 1:** Numbers of variables selected and model performance for ten automated methods of variable selection.

Technique	Number of variables in final model	Approach for evaluation of model performance	MAE	R^2^
Backward stepwise linear regression	265	Internal	26.5	0.95
		***Cross validation***	***136.0***	***0.28***
Multivariate adaptive regression splines	2	Internal	64.6	0.67
		***Cross validation***	***80.6***	***0.56***
Least absolute shrinkage and selection operator	36	Internal	57.0	0.73
		***Cross validation***	***64.6***	***0.65***
Ridge regression	335	Internal	56.0	0.78
		***Cross validation***	***74.8***	***0.57***
Elastic net	42	Internal	56.5	0.74
		***Cross validation***	***64.6***	***0.65***
Adaptive elastic net	3	Internal	63.6	0.68
		***Cross validation***	***70.3***	***0.58***
Smoothly clipped absolute deviation,	19	Internal	61.2	0.70
		***Cross validation***	***65.4***	***0.65***
Minimax convex penalty	3	Internal	65.0	0.67
		***Cross validation***	***67.4***	***0.63***
SparseStep	3	Internal	63.6	0.68
		***Cross validation***	***72.6***	***0.62***
Ranking-based variable selection	5	Internal	62.6	0.68
		***Cross validation***	***64.1***	***0.67***

**Figure 1:**
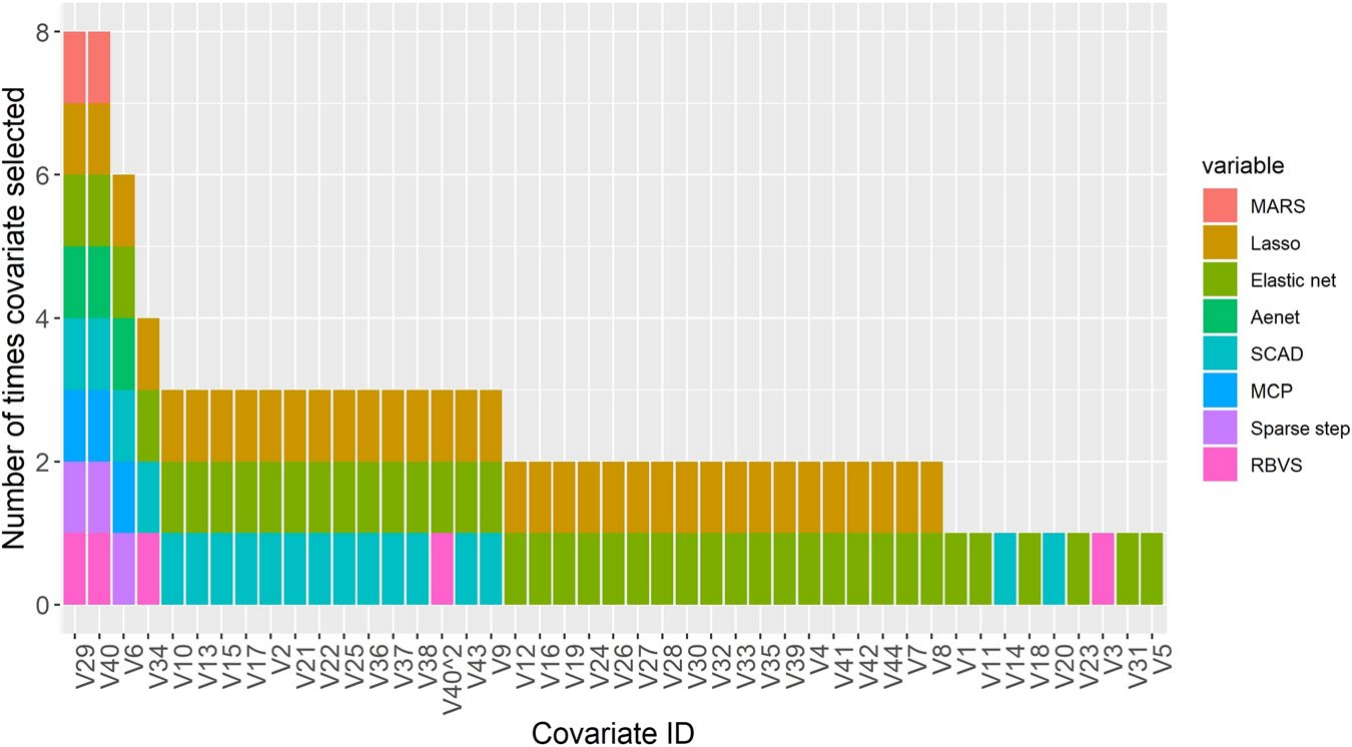
Covariates selected (out of 337 available) in final models using eight different automated methods of variable selection. Key; Sparse step – SparseStep regression, SCAD - smoothly clipped absolute deviation, Elastic net - elastic net regression, RBVS - ranking-based variable selection, MCP - minimax convex penalty, MARS - multivariate adaptive regression splines, Lasso - least absolute shrinkage and selection operator regression, Aenet - adaptive elastic net regression.

The covariates selected in each final model (excluding BSLR and ridge models in which the majority of covariates were selected and which were deemed to fit the data relatively poorly) are illustrated in Fig. 1 and coefficient estimates provided in Table 2. Despite similarity in cross validation MAE, considerable differences were identified in the actual covariates selected. Only two covariates were selected in all 8 models, these related to the type of housing system used by each study farm (V29) and the stocking density of animals on pasture (V40) and it was noticeable that these variables had the largest effect sizes. Two further variables were selected in at least half of the models and the other covariates were selected by 3 or fewer of the methods. However, of the variables selected in three or fewer models, the coefficients of some were sufficiently large to be of potential importance. For example, variables V3, V20, V36 and V43 all had coefficients of a magnitude to have a potentially important impact on the outcome, yet were selected in less than half of the final models. Therefore, results of these eight final models showed substantial heterogeneity in terms of the variables selected.

**Table 2:** Coefficients of variables selected in final models of eight automatic variable selection methods (blank spaces indicate the variable was not selected).

Variable ID	MARS	Lasso	Elastic net	Aenet	SCAD	MCP	Sparse step	RBVS
V29	58.5	34.8	35.7	54.0	45.7	34.6	58.6	59.2
V40	*207.2	181.5	182.2	191.7	196.2	198.4	19.2	218.7
V6		22.5	22.9	28.6	16.0	9.7	33.5	
V34		16.6	18.8		2.2			22.8
V2		5.4	6.2		0.6			
V9		8.4	8.3		5.0			
V10		2.7	4.4		7.0			
V13		6.5	7.8		2.2			
V15		12.2	13.2		3.6			
V17		2.3	3.9		3.6			
V21		8.3	10.0		3.1			
V22		3.2	2.7		1.6			
V25		13.5	14.0		5.7			
V36		−15.5	−15.9		−9.1			
V37		−7.9	−9.3		−0.7			
V38		12.1	12.9		2.3			
V43		21.7	22.9		11.5			
V40^2		2.2	0.3					−29.9
V4		2.4	3.0					
V7		9.3	9.8					
V8		2.3	3.0					
V12		1.0	2.1					
V16		−2.7	−2.6					
V19		−1.1	−2.2					
V24		4.8	4.2					
V26		−1.5	−2.7					
V27		0.2	0.7					
V28		1.2	2.4					
V30		−12.6	−14.5					
V32		−4.5	−6.4					
V33		−0.5	−1.4					
V35		−0.6	−1.6					
V39		3.3	4.6					
V41		2.1	2.5					
V42		−7.4	−8.0					
V44		0.8	2.5					
V1			0.5					
V3								39.7
V5			−0.6					
V11			1.7					
V14					4.5			
V18			0.2					
V20					−22.4			
V23			0.7					
V31			−0.5					

*Represents a hinge function of variable V40.

Key; Sparse step – SparseStep regression, SCAD - smoothly clipped absolute deviation, Elastic net - elastic net regression, RBVS - ranking-based variable selection, MCP - minimax convex penalty, MARS - multivariate adaptive regression splines, Lasso - least absolute shrinkage and selection operator regression, Aenet - adaptive elastic net regression.

### Selection stability results

**Figure 2:**
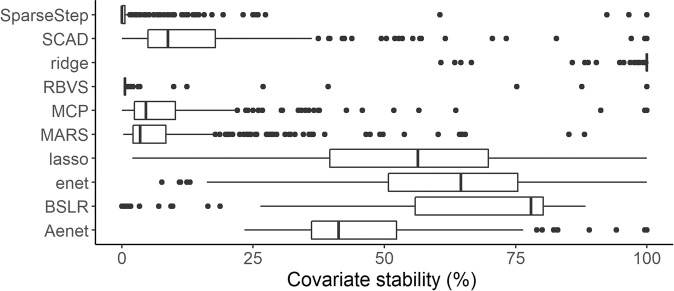
Illustration of the distribution of covariate selection stability for ten methods of automated covariate selection. Selection stability was defined as the percentage of bootstrap samples (out of 500) that each covariate (n = 337) was selected by each specified method. Key; SparseStep – SparseStep regression, SCAD - smoothly clipped absolute deviation, ridge - ridge regression, RBVS - ranking-based variable selection, MCP - minimax convex penalty, MARS - multivariate adaptive regression splines, lasso - least absolute shrinkage and selection operator regression, BSLR - backward stepwise linear regression, enet - elastic net regression, Aenet - adaptive elastic net regression.

Five hundred bootstrap samples were used to estimate covariate stability and stability varied considerably between methods (Fig. 2). The ridge method consistently retained virtually all variables in all bootstrapped samples, producing a non-sparse model with a large number of variables being retained in 100% of samples. For all other methods, relatively few covariates were selected in >90% of bootstrap samples; the range was 1 variable (RBVS) to 24 (elastic net). Covariates that had a high stability (>90%) in at least one model (excluding BSLR and ridge), are listed in Table 3, and a comparison is provided of the stability of these covariates between methods. It was evident that stability varied greatly between methods; several variables (e.g. V37, V19) could have a stability of >90% using one method and <10% using another. Of the 24 variables that had a stability >90% in at least one method, nine of these (X1 – X9) were variables that had not been selected in any of the original non-bootstrapped final models.

**Table 3:** Maximum and median selection stability values (%) for covariates across eight statistical models, ranked in order of median stability. Covariates shown all had a stability >90% in at least one method (BSLM and ridge methods excluded).

Variable ID	Maximum Stability	Median Stability	SCAD	Lasso	MARS	MCP	Aenet	Enet	RBVS	SparseStep
V40	100	100	100	100	65	100	100	100	99	100
V29	100	97	97	100	54	91	100	100	60	97
V34	100	79	71	100	88	52	94	100	29	27
V6	92	73	83	90	39	64	89	92	20	61
V36	92	65	73	92	47	57	83	90	0	12
V42	95	51	42	91	60	30	83	95	0	7
V39	95	48	49	95	36	46	80	94	10	17
V21	93	46	57	90	20	34	83	93	2	16
V37	94	45	53	91	37	35	62	94	0	10
V10	94	44	52	92	20	37	82	94	2	7
V30	98	40	32	98	47	22	64	98	0	10
X1	92	38	23	88	65	12	53	92	1	1
V2	92	36	42	90	12	31	71	92	3	26
V4	90	31	36	88	7	21	61	90	1	26
X2	93	28	20	91	36	17	54	93	0	0
V19	90	27	32	88	4	21	65	90	0	0
X3	95	25	29	91	7	20	66	95	0	1
V8	91	25	26	84	4	17	57	91	1	23
X4	99	22	27	97	10	18	66	99	1	14
V41	95	20	15	92	25	6	74	95	13	5
X5	92	19	9	83	29	7	42	92	0	0
X6	93	18	11	90	25	9	61	93	0	0
X8	92	13	15	87	5	10	61	92	0	0
X9	92	7	10	87	2	5	47	92	0	2

Key; SparseStep – SparseStep regression, SCAD - smoothly clipped absolute deviation, Enet - elastic net regression, RBVS - ranking-based variable selection, MCP - minimax convex penalty, MARS - multivariate adaptive regression splines, Lasso - least absolute shrinkage and selection operator regression, Aenet - adaptive elastic net regression

The median stability was calculated for each covariate across eight selection methods (excluding BSLR and ridge which from the initial models, were deemed to fit too poorly to use for subsequent inference). Six of the 337 variables had a median stability >50% and of these only two had a median stability >90%. The two variables with the highest median stability, V40 and V29, were the variables selected by all methods when the original non-bootstrapped models were constructed (Fig. 1).

Three conventional linear models were fit using subsets of covariates with relatively high bootstrap stability across all methods and 10×10 fold cross validation conducted to evaluate model performance. A linear model built using the two variables with a median stability >90% (variables V40 and V29, Table 2) resulted in an internal MAE = 65.0 and R^2^ = 0.67 and a cross validation MAE = 65.9 and R^2^ = 0.65. When six variables with a median stability across methods >50% were modelled, the resulting internal model fit metrics were MAE = 62.3 and R^2^ = 0.69, and cross validation fit metrics MAE = 65.0 and R^2^ = 0.66. A further linear model built with covariates that had a bootstrap stability of >90% in any of the methods (n = 24 covariates, Table 2) provided the best overall cross validation performance and better than any of the original ten methods implemented, with an internal MAE = 56.0, R^2^ = 0.75 and a cross validation MAE = 61.4, R^2^ = 0.70. Coefficients and confidence intervals for this model, including for comparison, bootstrap confidence intervals from the initial models, are provided in Supplementary Information (Table S1).

The ranking of covariate stability was explored between all ten automated model selection methods using non-parametric correlation analysis, results are presented in Table 4. Spearman correlation coefficients >0.6 existed between the following methods i) SCAD, MCP and adaptive elastic net, and ii) Elastic net, lasso and SparseStep. Covariate stability of MARS showed a degree of similarity to SCAD, Lasso, MCP and elastic net (Spearman correlations 0.44 to 0.50) but covariate stability of ridge and BLSR showed little correlation with other methods.

**Table 4:** Spearman correlations between variable selection stability by method.

Stability	SCAD	Lasso	MARS	MCP	Ridge	Aenet	Enet	RBVS	Sparse step	BSLR
SCAD		0.50	0.46	0.93	−0.05	0.84	0.48	0.49	0.50	0.05
Lasso	0.50		0.45	0.52	0.39	0.47	0.98	0.37	0.60	0.09
MARS	0.46	0.45		0.50	0.15	0.39	0.44	0.41	0.38	−0.27
MCP	0.93	0.52	0.50		0.00	0.85	0.51	0.49	0.50	0.07
Ridge	−0.05	0.39	0.15	0.00		−0.08	0.39	0.07	0.19	0.15
Aenet	0.84	0.47	0.39	0.85	−0.08		0.46	0.48	0.39	0.04
Enet	0.48	0.98	0.44	0.51	0.39	0.46		0.38	0.59	0.08
RBVS	0.49	0.37	0.41	0.49	0.07	0.48	0.38	1.00	0.53	−0.04
Sparse step	0.50	0.60	0.38	0.50	0.19	0.39	0.59	0.53		0.01
LM	0.05	0.09	−0.27	0.07	0.15	0.04	0.08	−0.04	0.01	

Key; Sparsestep – SparseStep regression, SCAD - smoothly clipped absolute deviation, Ridge - ridge regression, RBVS - ranking-based variable selection, MCP - minimax convex penalty, MARS - multivariate adaptive regression splines, Lasso - least absolute shrinkage and selection operator regression, BSLR - backward stepwise linear regression, Enet - elastic net regression, Aenet - adaptive elastic net regression.

## Discussion

Results of this research illustrate that choice of method can have a substantial influence on the subset of variables selected in modelling relatively high dimensional data and that triangulation of results across methods can greatly enhance data interpretation and confidence in variable selection The ten methods of automated variable selection used to model these data produced markedly different results, which meant if used alone, would lead to different conclusions being drawn from the same data and different variables being carried forward for future study. It is rare that more than one technique is used when conducting high-dimensional analyses, but these results align with the view that evaluation of multiple methods is a useful strategy to ensure that uncertainty in data is considered from multiple angles^15,17^. Indeed, it has been argued that in the face of a ‘reproducibility crisis’ in research^18^, use of multiple analytic approaches to fully explore data and identify robust solutions may partly mitigate problems with reproducibility^19^. Our results support this view and use of multiple analytic approaches added valuable insights to relationships within these data. For example, two variables were selected by all methods and had relatively large effect sizes; this triangulation provided confidence that these variables were likely to be the most important of the 377 available, which was not clear from use of any individual method. Quantification of selection stability across all methods provided further insights. Firstly, the two covariates, V29 and V40, that were selected by all methods initially, were also the most stable across all methods (median stability >90%); this provided further evidence for the likely importance of these variables. A linear model fit using solely these two variables demonstrated that they explained a large proportion of the variation in the outcome (R^2^ = 0.65) compared to that explained by the best model (R^2^ = 0.70), again providing confidence in their relative importance in these data.

After identification of these two key variables, use of selection stability across methods also aided the detection of further covariates of potential importance that could warrant further evaluation**;** using the median and maximum stability across methods, new subsets of variables were identified. In fact, the combination of 24 covariates identified as being highly stable (>90%) in at least one of the methods resulted in a linear model with the best overall cross validation performance characteristics. This combination of variables was not identified by any of the individual methods but in terms of minimising the cross validation MAE could be described as the best model for these data, again indicating the usefulness of employing multiple analytic approaches. The coefficient distributions (Table S1, Supplementary Information) provided further information to evaluate and rank these 24 variables in terms of relative importance; those with confidence intervals furthest from zero across many methods deemed most likely to be important and worthy of follow up. Whilst the choice and number of variables to follow up from a particular study will depend on resources available as well as the effect size and plausibility of each variable, this approach using between method selection stability provided a useful framework to inform such decisions.

It was notable that there were marked differences in the sparseness of final models between methods; some variables with apparently relatively large effect sizes were selected by some methods and not others. Backward stepwise linear regression and ridge regression produced the least sparse models and poor results in terms of discrimination of important variables and cross validation fit characteristics. These methods were discounted as being useful for variable selection with these data and confirms that stepwise regression is generally considered unsuitable for variable selection with high dimensional data^1^. Comparison of variable selection between methods was useful because a rounded evaluation could be made from more and less sparse models. Variables with high stability in any method could be considered of potential importance and since stability varied between methods, this allowed a subset of variables to be identified that had not been selected by any individual method.

The correlations in bootstrap selection stabilities between different models indicated some similarity between the variable selection methods used. Despite the fact that MCP produced a sparser solution than SCAD, the two methods were highly correlated in terms of ranking of variable stability (Spearman correlation 0.98). This may not be surprising since the methods of regularisation employed have similarity (Eqs. 7–9); both of these methods incorporate a non-linear penalisation and apply less shrinkage with increasing size of coefficient^20,21^. Similarly, both SCAD and MCP had selection stability rankings highly correlated to Aenet, another method that incorporates reduced penalisation with increasing size of coefficient^22^. Elastic net and lasso also produced highly correlated selection stabilities, again this reflects similarities of these methods. Whilst lasso is based on the conventional L_1_ regularisation, a single penalty applied to the sum of the absolute coefficient values (Eq. 3), elastic net combines this with the L_2_ penalty, a penalty applied to the sum of the squared coefficient values (Eq. 5).

There was a notable contrast in the degree of covariate stability between modelling methods. Of the most sparse models, SparseStep and MCP identified 2 covariates as being >90% stable out of the three variables originally selected in the full models. RBVS identified one covariate as >90% stable of the original 5 and MARS identified neither of the two variables originally selected as >90% stable. In contrast, for Aenet, all three of the originally selected variables had >90% stability. Of the less sparse models, out of 19 variables originally selected using SCAD, only 3 were >90% stable whereas both lasso and elastic net identified a relatively large number of variables as being >90% stable. The variability in stability between methods suggests some methods are more inclined to produce different results under perturbations of the data than others and confirms the view that selection stability is a crucial addition to the variable selection process^6,9^.

In conclusion, the use of different statistical methods to select a sparse set of important variables resulted in very different subsets of variables being identified. Evaluation of multiple methods and selection stability provided invaluable insight to aid variable selection in these epidemiological relatively high-dimensional data. These findings indicate that use of triangulation of results across methods can greatly enhance data interpretation and confidence in variable selection.

## Materials and Methods

### Data collection and preparation

Data for the study came from previous research conducted on 408 commercial sheep farms in the UK^23^. The original study aim was to identify covariates associated with increased farm income per acre, to determine the best candidates for intervention studies to improve farm profitability. Data collection and pre-processing have been described in detail previously^23^; a brief overview is provided below.

The outcome variable of interest was farm revenue (£) derived from lamb sales per unit area farmed (acre) for the year 2017. This variable was approximately normally distributed with a median £197 per acre (IQR £120–£297). The potential explanatory covariates comprised information on farmer demographics, farm management strategies and farmer attitudes, and were collected by questionnaire. Farms were based in the UK; 76% were located in Wales, 18% in England and 4% and 2% in Scotland and Northern Ireland respectively. The median farm size was 265 acres (IQR 150–450) and the median flock size 560 breeding ewes (IQR 329–873).

A total of 337 explanatory variables were available and following imputation of a small number of data points, there were no missing values in the final dataset. Whilst the precise details of the covariates are not relevant to this research, full details can be found in Lima *et al*.^23^. Specific potential confounding variables such as flock size were included in the model selection. Continuous variables (n = 42) were centred and standardised by two standard deviations to allow direct comparisons to be made between model coefficients^24^. Six continuous covariates were included as polynomial terms up to power four because non-linear relationships with the outcome were suspected.

### Analytics

Ten commonly used statistical approaches that incorporate automated covariate selection were employed to analyse the data. Identical data were used for each statistical method; the outcome variable was farm revenue per acre and all 337 covariates were included as explanatory variables. A common approach to implementation of each method was used as follows. Firstly, each model was fit to the data and, where required, model hyperparameters optimised using ten-fold cross validation repeated ten times. For each method, a ‘final’ model was selected using hyperparameter values that resulted in the lowest cross validation mean absolute error (MAE); the selected covariates and coefficients at the optimised hyperparameter value were identified. Subsets of selected covariates in each final model were compared between methods.

To evaluate the extent of over- or under-fitting in the final models, a comparison was made between the MAE and R^2^ computed for each final model using the full dataset (‘internal fit’) and those calculated from 10 × 10 fold cross validation (‘cross validation fit’).

To further assess between model heterogeneity in covariate selection, covariate selection stability was evaluated for all models. Selection stability is a concept well described in the context of model selection^7,9,10^; the basis is to evaluate the extent to which covariate selection changes under perturbations of the data. The most stable variables are the ones least likely to change when the data are perturbed and therefore can be considered most likely to have an effect across largest parts of the data and in other similar populations. In this case we evaluated covariate stability for each model using a bootstrapping methodology. We estimated covariate stability for each method, as the percentage of times that each covariate was selected in the model across 500 bootstrap samples. The distribution of covariate coefficients were also calculated from all non-zero (i.e. variables that were selected) values of the coefficient in the bootstrap samples. Therefore, we not only compared covariates selected between the ten different statistical methods used, but for each method, we also calculated the stability of variable selection. This allowed comparisons between all models of the most stable variables (for example, those with a stability of ≥90%) and the extent to which the most stable variables were similar between methods.

The ten methods used for analysis were; backward stepwise linear regression (BSLM)^25^, multivariate adaptive regression splines^26^, least absolute shrinkage and selection operator regression^11^, ridge regression^12^, elastic net regression^11,13^, adaptive elastic net regression^27^, smoothly clipped absolute deviation^20^, minimax convex penalty^21^, Sparsestep^28^, and ranking-based variable selection^29^. These approaches are summarised below; all models were run using the R statistical framework^30^.

### Backward Stepwise Linear Regression (BSLR)

A conventional linear regression model was implemented and can be described as;

1 $$y={\beta }_{0}+\mathop{\sum }\limits_{j=1}^{p}{\beta }_{j}{x}_{j}+e$$

where $y$ is the response variable, ${\beta }_{0}$ an intercept term, ${x}_{j}$ represents the j^th^ of $p$ covariates with an estimated coefficient ${\beta }_{j}$, $e$ is the residual model error. Covariate selection was conducted using a backward stepwise procedure with minimisation of the Akaike information criterion (AIC) as the loss function. The AIC is defined as 2 ${\rm{k}}-2\,\mathrm{ln}(\hat{{\rm{L}}})$ where k is the number of parameters in the model and $\hat{{\rm{L}}}$ the likelihood function. The BSLR model was estimated using the MASS package in R^25^. Covariate stability was evaluated from 500 bootstrap samples using the R package bootstepAIC^31^.

### Multivariate adaptive regression splines

Multivariate adaptive regression spline (MARS) models^26^ are a flexible form of regression modelling that perform automatic variable selection as well as identification of non-linearities and interactions. Non-linear functions are represented by hinge functions^26^ and the MARS model can be described as;

2 $$y={\beta }_{0}+\mathop{\sum }\limits_{j=1}^{p}{\beta }_{j}{h}_{j}{x}_{j}+e$$

where $y,\,{\beta }_{0}$, *j*, $p$ and $e$ are as defined in (1), ${h}_{j}\,{x}_{j}$ is a function of covariate *x*_*j*_ or a product of two or more such functions, with coefficient $\,{\beta }_{j}$. MARS uses expansions in linear basis functions which are generally specified as (x − t)+ and (t − x)+ (where “+” indicates the positive part); each function is piecewise linear, with a knot at the value t. These ‘hinge’ functions and can be represented by;

$$\begin{array}{cccc}(x-t)+ & = & {\rm{x}}-{\rm{t}}, & {\rm{i}}{\rm{f}}\,{\rm{x}} > {\rm{t}}\\  &  & 0, & {\rm{i}}{\rm{f}}\,{\rm{x}}\le {\rm{t}}\end{array}$$

and

$$\begin{array}{cccc}(t-x)+ & = & {\rm{t}}-{\rm{x}}, & {\rm{i}}{\rm{f}}\,{\rm{x}} > {\rm{t}}\\  &  & 0, & {\rm{i}}{\rm{f}}\,{\rm{t}}\le {\rm{x}}\end{array}$$

Model selection is made firstly by using a forward iterative procedure to identify the combination of hinge functions and interactions that minimise the least squares error followed by a backward deletion step (‘pruning’) in which model terms that produce the smallest increase in residual squared error are deleted from the model. Hyperparameters optimised using 10 ×10 fold cross validation were “nprune”, the maximum number of terms (including intercept) allowed in the pruned model and “degree”, the maximum degree of interactions incorporated in the model. MARS models were constructed using the earth package^32^ within the caret package platform^33^ in R.

### Least absolute shrinkage and selection operator regression

A least absolute shrinkage and selection operator (lasso) model^11^ was the first of several regularised modelling approaches implemented with the data. The others, ridge regression, elastic net, adaptive elastic net, smoothly clipped absolute deviation, minimax convex penalty and Sparsestep, are described below. The general principle of regularised approaches, which are an extension of the linear regression Eq. (1), is that a penalty is applied to covariate coefficients to shrink them towards zero and to set some to exactly zero. Whilst this increases model bias, it can be associated with a reduction in variance and improved model fit^34^. In the case of Lasso, the penalty is bound to the sum of the absolute values of the coefficients (L_1_ penalty) and the lasso loss function can be represented;

3 $${\rm{SSE}}\_{\rm{lasso}}=\mathop{\sum }\limits_{i=1}^{n}{({y}_{i}-{\hat{y}}_{{\rm{i}}})}^{2}+{{\rm{\lambda }}}_{L}\mathop{\sum }\limits_{j=1}^{p}|{\beta }_{j}|$$

where SSE_lasso represents the lasso loss function to be minimised, i denotes each observation and n the number of observations, y_i_ and ŷ_i_ are respectively the observed and the predicted outcome for the ith observation, j denotes a predictor variable with p the number of predictor variables in total, and |β| represents absolute values of the regression coefficients. The optimal value of λ_L_, the penalisation hyperparameter, was determined as that producing the lowest MAE using 10 ×10 fold cross validation.

### Ridge regression

Ridge regression^12^ is an alternative form of regularised regression in which a penalty is applied to the square of the coefficients (L_2_ penalty). The ridge loss function takes the form;

4 $${\rm{SSE}}\_{\rm{ridge}}=\mathop{\sum }\limits_{i=1}^{n}{({y}_{i}-{\hat{y}}_{{\rm{i}}})}^{2}+{{\rm{\lambda }}}_{R}\mathop{\sum }\limits_{j=1}^{P}|{\beta }_{j}^{2}|$$

where SSE_ridge represents the ridge loss function to be minimised, and i, y_i_, ŷi, j, p and n are all defined as in Eq. (3). The optimal value of λ_R_, the penalisation hyperparameter, was determined by 10×10 cross validation.

### Elastic net regression (Enet)

Elastic net is a combination of lasso and ridge regression and incorporates both the L_1_ and L_2_ penalties and can be represented as;

5 $$SS{E}_{enet}=\frac{1}{2n}{\sum }_{i=1}^{n}{({y}_{i}-{\hat{y}}_{{\rm{i}}})}^{2}+{\lambda }_{E}\,\left[{\sum }_{j=1}^{P},(,\frac{1}{\,2},(,1,-,\alpha ,),{\beta }_{j}^{2},+,\alpha ,|,{\beta }_{j},|\right]$$

where SSE_enet_ represents the elastic net loss function to be minimised, i, y_i_, ŷ_i_, j, p and n are as defined in Eq. (3). The hyperparameters that represent the penalty ${({\rm{\lambda }}}_{E})$ and the relative proportion of penalisation on either the sum of the square of the coefficients or the unsquared coefficients (α) were optimised by 10 ×10 fold cross validation again to minimise MAE.

The lasso, ridge and elastic net models were built using the glmnet package  in the caret package platform^33^ in R^30^.

### Adaptive elastic net regression (Aenet)

The adaptive elastic net is an extension of the elastic net such that the lasso (L1) component of penalty is modified to a weighed (adaptive) lasso penalty^27^. In the adaptive lasso, variables with larger coefficients are assigned smaller weights and the extent of differential penalty weightings is a hyperparameter. The adaptive elastic net loss function can be described in terms of the elastic net loss function (3) but with an additional weighting factor, w, applied to each covariate coefficient, which is dependent on the size of the coefficient (β) as follows;

6 $$SS{E}_{aenet}=\frac{1}{2n}{\sum }_{i=1}^{n}{({y}_{i}-{\hat{y}}_{{\rm{i}}})}^{2}+{{\rm{\lambda }}}_{E}\,\left[{\sum }_{j=1}^{P}(\frac{1}{\,2}(1-{\rm{\alpha }}){\beta }_{j}^{2}+\alpha {w}_{j}|{\beta }_{j}|\right]$$

where SSE_aenet_ represents the adaptive elastic net loss function to be minimised, all other model parameters are as defined in Eq. (3), and w is defined as;

$${w}_{j}=|{\beta }_{jenet}{|}^{-\gamma }$$

where $|{\beta }_{jenet}|$ are the absolute coefficient values derived from an initial elastic net model defined in (3) and $\gamma$ is a hyperparameter optimised by 10 × 10 fold cross validation. Adaptive elastic net regression was conducted in the R package msaenet^22^.

### Smoothly clipped absolute deviation and minimax convex penalty

Smoothly clipped absolute deviation (SCAD)^20^ and minimax convex penalty (MCP)^14,21^ are additional related forms of regularised regression. A key feature of these methods is that, as with adaptive elastic net, the size of the penalty function varies with the size of the covariate coefficient, β. Both methods can be described by the general framework;

7 $$SS{E}_{scad/mcp}=\,{\sum }_{i=1}^{n}{({y}_{i}-{\hat{y}}_{{\rm{i}}})}^{2}+\mathop{\sum }\limits_{j=1}^{p}P({\beta }_{j}|{\rm{\lambda }},\gamma )$$

where SSE_scad/mcp_ represents the SCAD or MCP loss function to be minimised, i, y_i_, ŷ_i_, j, p and n are as defined in Eq. (3) and $P({\beta }_{j}|{\rm{\lambda }},\gamma )$ represents a penalty function as follows;

For SCAD:

8 $$\begin{array}{cccc}P(\beta |{\rm{\lambda }},\gamma ) & = & {\rm{\lambda }}, & {\rm{if}}|{\rm{\beta }}|\le {\rm{\lambda }}\\  &  & \frac{\gamma {\rm{\lambda }}-\,|{\rm{\beta }}|}{\gamma -1}, & {\rm{if}}\,{\rm{\lambda }} < |{\rm{\beta }}| > \gamma {\rm{\lambda }}\end{array}$$

$$0,\,{\rm{if}}|{\rm{\beta }}|\ge \gamma {\rm{\lambda }}$$

For MCP:

9 $$\begin{array}{cccc}P(\beta |{\rm{\lambda }},\,\gamma ) & = & {\rm{\lambda }}|{\rm{\beta }}|-\frac{{\beta }^{2}}{2\gamma }, & {\rm{if}}|{\rm{\beta }}|\le \gamma {\rm{\lambda }}\\  &  & 0.5\gamma {{\rm{\lambda }}}^{2} & {\rm{if}}\,|{\rm{\beta }}| > \gamma {\rm{\lambda }}\end{array}$$

where ${\rm{\gamma }}\,{\rm{and}}\,{\rm{\lambda }}$ are hyperparameters optimised using 10 × 10 fold cross validation. Both SCAD and MCP models were estimated using the R package ncvreg^35^. The non-linear penalties applied by the SCAD and MCP techniques mean, as with adaptive elastic net, relatively less shrinkage is applied as the absolute size of coefficients increases.

### SparseStep

The SparseStep function has been relatively recently described and provides another approach for non-linear penalisation in the regression loss function^28^. The SparseStep loss function can be described as;

10 $$SS{E}_{sp\_step}={\sum }_{i=1}^{n}{({y}_{i}-{\hat{y}}_{{\rm{i}}})}^{2}+{\rm{\lambda }}{\sum }_{j=1}^{p}\frac{{\beta }^{2}}{{\beta }^{2}+{\gamma }^{2}}$$

where SSE_sp_step_ represents the SparseStep loss function to be minimised, i, y_i_, ŷ_i_, j, p and n are as defined in Eq. (3) and $\lambda \,{\rm{and}}\,\gamma$ are hyperparameters optimised using 10 ×10 fold cross validation. The Sparsestep model was estimated using the sparsestep package in R^28^.

### Iterative ranking-based variable selection

Iterative ranking-based variable selection (RBVS) is a different approach to that of regularisation, for the identification of sparse models. The theory and methods of estimation have been described in detail^29^ and we provide an overview of the concepts. RBVS is based on the principle that truly important covariates will consistently be related to an outcome of interest, both over an entire sample and over randomly chosen sample subsets. RBVS uses a method of ranking variables in terms of their association with the outcome and this is repeatedly conducted over many subsamples of the data. A set of top ranked variables is identified and removed from the dataset and the procedure repeated at a second iteration to identify the next top ranked set. Iterations are continued until no further top sets of variables are identified. RBVS was conducted using the R-package “rbvs”^36^ with lasso regression used as the method for variable ranking. The size of subsample used was 200 and 100 repeated samples were used at each iteration to identify the top set of variables. The maximum number of variables allowed in the subset of important variables at each iteration was set at 10. The top ranked variables identified were deemed to comprise a ‘final model’ and coefficients for these variables were estimated using a conventional linear regression model with and 10 ×10 fold cross validation used to estimate cross validation MAE and R^2^.

## Supplementary information

Supplementary information.
