# Supplementary material for: Variable selection for inferential models with relatively high-dimensional data: Between method heterogeneity and covariate stability as adjuncts to robust selection
Source: Sci Rep. 2020 May 14;10:8002. doi: 10.1038/s41598-020-64829-0 (PMC7224285; doi:10.1038/s41598-020-64829-0)
Supplement: Supplementary file 1 — Supplementary information. [file 41598_2020_64829_MOESM1_ESM.pdf]

Variable selection for inferential models with relatively high-dimensional data: Between method heterogeneity and covariate stability as adjuncts to robust selection

Author names and affiliations

Eliana Lima[1, 3], Peers Davies[2], Jasmeet Kaler[1], Fiona Lovatt[1], Martin Green*[1]

[1] School of Veterinary Medicine and Science, University of Nottingham, Sutton Bonington Campus, Leicestershire, LE12 5RD, United Kingdom

[2] Department of Epidemiology and Population Health, Institute of Infection and Global Health, University of Liverpool, Liverpool, L69 7BE, United Kingdom

[3] Current address: OIE, World Organisation for Animal Health 12, rue de Prony, 75017 Paris, France

*** Corresponding author**

Martin Green

E-mail: martin.green@nottingham.ac.uk

Supplementary Information

Variable ID	LM Coef	LM 95% CI	Lasso 95% BI	Enet 95% BI	Aenet 95% BI	SCAD 95% BI	MCP 95% BI	Sparsestep 95% BI	RBVS 95% BI
**V40**	182	161...202	111...227	65...223	67...206	154...247	146...250	164...237	187...248
**V39**	59	39...78	0...67	3...69	0...54	0...69	0...69	29...63	23...33
**X2**	49	23...74	0...72	0...67	0...53	0...67	0...64		
**V29**	46	24...67	14...82	12...80	13...76	0...90	0...92	35...92	56...71
**X1**	45	24...67	1...80	0...79	0...50	0...55	0...58	28...49	14...47
**X9**	36	13...59	1...64	1...62	0...40	0...12	0...21	29...66	
**V34**	36	17...55	12...73	12...77	5...65	0...68	0...75	18...74	17...25
**V30**	-32	-52...-12	-155...-6	-126...-4	-66...0	-79...0	-78...0	-79...-16	
**V10**	31	5...56	1...78	1...77	0...61	0...50	0...61	38...74	33...43
**X4**	29	12...47	1...53	3...54	0...34	0...21	0...33	16...41	10...11
**V6**	25	8...42	-5...39	-5...40	1...40	0...51	0...49	23...54	29...43
**V36**	-24	-41...-7	-42...4	-43...7	-36...-1	-44...0	-47...0	-50...-25	
**V21**	20	0...41	0...48	1...54	0...47	0...45	0...48	29...56	25...29
**X5**	20	2...38	-3...48	-2...55	-7...39	-4...2	-3...16		
**X8**	-18	-38...2	-63...0	-59...0	-48...0	-18...0	-35...0		
**V37**	-18	-34...-1	-38...3	-43...8	-29...0	-33...0	-34...0	-44...-25	
**V8**	18	0...35	-3...40	-2...45	0...28	0...16	0...22	16...56	
**X3**	-17	-40...5	-63...-1	-60...0	-47...0	-42...0	-43...0	-63...-38	
**V2**	17	-1...34	-1...46	-1...44	0...40	0...35	0...42	19...47	16...25
**V19**	-15	-31...0	-40...1	-47...1	-32...0	-18...0	-29...0		
**V4**	13	-3...30	-2...39	-6...36	0...28	0...26	0...22	18...43	18...26
**V42**	-11	-27...6	-85...6	-77...5	-123...-1	-133...0	-137...0	-161...-22	
**V41**	11	-10...31	-3...64	1...71	2...49	0...41	0...46	23...59	17...39
**X6**	-6	-23...11	-51...-1	-51...0	-38...0	-10...0	-29...0	-26...-22	

Key; LM – conventional ordinary least squares linear regression, Sparsestep – SparseStep regression, SCAD - smoothly clipped absolute deviation, Ridge - ridge regression, MCP - minimax convex penalty, Lasso - least absolute shrinkage and selection operator regression, Enet - elastic net regression, Aenet - adaptive elastic net regression, RBVS - ranking-based variable selection.

Table S1. Coefficient estimates and 95% confidence intervals(CI) of variables included in a convention linear model (LM) that produced the best cross validation fit to the study data. Variables were identified from having ≥90% stability in at least one of ten automated covariate selection methods. The 95% bootstrap probability intervals (BI) are also provided for the same variables calculated from the 500 bootstrapped coefficient values obtained during bootstrapping of the original individual variable selection models. (Gaps are where variables were selected in less than 1% of bootstrap samples for that method).
